# CD4 cell count and the risk of infective and non-infective serious non-AIDS events in HIV-positive persons seen for care in Italy

**DOI:** 10.7448/IAS.17.4.19509

**Published:** 2014-11-02

**Authors:** Giordano Madeddu, Antonella D'Arminio Monforte, Enrico Girardi, Antonio Di Biagio, Sergio Lo Caputo, Roberta Piolini, Giulia Marchetti, Giampietro Pellizzer, Andrea Giacometti, Laura Galli, Andrea Antinori, Alessandro Cozzi Lepri, on behalf of the ICONA Foundation Study

**Affiliations:** 1 Unit of Infectious Diseases, University of Sassari, Sassari, Italy; 2 Health Sciences, San Paolo Hospital, University of Milan, Milan, Italy; 3 Clinical Epidemiology, National Institute for Infectious Diseases, Rome, Italy; 4 Infectious Diseases, IRCCS San Martino Hospital, Genoa, Italy; 5 Infectious Diseases, Santa Maria Annunziata Hospital, Florence, Italy; 6 Infectious Diseases, Luigi Sacco Hospital, University of Milan, Milan, Italy; 7 Infectious Diseases, Vicenza Hospital, Vicenza, Italy; 8 Infectious Diseases, Ancona Hospital, Ancona, Italy; 9 Infectious Diseases, San Raffaele Hospital, Milan, Italy; 10 Clinical Department, National Institute for Infectious Diseases “L. Spallanzani”, Rome, Italy; 11 Infection and Population Health, University College London, London, UK; 12 Health Sciences, University of Milan, Milan, Italy

## Abstract

**Introduction:**

Serious non-AIDS events (SNAE) are frequent in HIV patients receiving cART. Current CD4 count was shown to be more strongly associated with infective compared to not-infective SNAE and unable to predict cardiovascular events. We investigated the relationship between baseline and current CD4 count and the risk of both infective and non-infective SNAE in HIV-positive patients according to current ART use.

**Methods:**

We included all HIV-positive persons enrolled in the ICONA Foundation Study cohort who had at least one follow-up visit. SNAE were grouped in infective (pneumonia, sepsis, endocarditis and meningitis) and non-infective (malignancies, chronic kidney disease, cardiovascular events, hepatic events and pancreatitis) aetiology. Incidence of these event groups were calculated overall and according to baseline and current CD4 count (grouped as 0–200, 201–350, 351–500, 501–750, and >750 cells/mm^3^). Participants’ follow-up accrued from the date of enrolment (baseline) to a diagnosis of SNAE or their last visit. An event was defined the first time one of the considered SNAE occurred so that each person contributed a single event. A Poisson regression model for each of the two endpoints was used.

**Results:**

A total of 10,822 patients were included (25.3% females, 38.2% heterosexuals) and 26.6% had hepatitis co-infections. Median age was 36 (IQR 31–42) years. Overall, 423 not-infective and 385 infective SNAE were included. The most frequent non-infective SNAE were malignancies (*n*=202) and the most frequent infective SNAE were pneumonia (*n*=289). Crude rates of non-infective SNAE were 0.78, 1.08 and 0.80/100 PYFU, and those of infective SNAE were 1.00, 0.51 and 0.66/100 PYFU in ART naive, currently off and currently on ART patients, respectively. Higher current CD4 count was associated with reduced risk of both infective and non-infective SNAE in naives and in patients on ART (Table 1). The association was less strong in the group which suspended ART (for non-infective SNAE the *p* value for interaction between current log-CD4 and ART-status, *p*=0.004). Conversely, we found no association between baseline CD4 count and risk of non-infective SNAE in people treated with ART (*p* value for interaction = 0.0001). When CVD were considered separately, there was no association with CD4 count (not shown).

**Conclusions:**

Our findings show that, differently from ART naive, in ART-treated patients, non-infective SNAE are predicted by current but not by baseline CD4, suggesting that immune restoration is crucial to prevent these events.

**Table 1:** Adjusted rate ratios (ARR) of infective and non-infective serious non-AIDS events (SNAE) from fitting Poisson regression models

		Infective SNAE			Non-infective SNAE	
						
	ART naive	Currently off ART	Currently on ART	ART naive	Currently off ART	Currently on ART
	
	ARR (95% CI), *p* value	ARR (95% CI), *p* value	ARR (95% CI), *p* value	ARR (95% CI), *p* value	ARR (95% CI), *p* value	ARR (95% CI), *p* value
Current CD4 count (cells/mmc)						
≤200	1.00	1.00	1.00	1.00	1.00	1.00
201–350	0.19 (0.10, 0.34), <0.001	1.00 (0.28, 3.55), 0.996	0.35 (0.23, 0.52), <0.001	0.40 (0.20, 0.81), 0.011	0.90 (0.33, 2.47), 0.832	0.48 (0.32, 0.72), <0.001
351–500	0.11 (0.06, 0.20), <0.001	0.38 (0.08, 1.83), 0.226	0.24 (0.16, 0.37), <0.001	0.18 (0.09, 0.36), <0.001	0.55 (0.19, 1.56), 0.258	0.56 (0.37, 0.84), 0.005
501–750	0.08 (0.04, 0.16), <0.001	0.10 (0.01, 0.86), 0.036	0.21 (0.13, 0.32), <0.001	0.19 (0.10, 0.35), <0.001	0.26 (0.07, 0.93), 0.027	0.47 (0.33, 0.71), <0.001
>750	0.11 (0.05, 0.22), <0.001	0.20 (0.01, 2.06), 0.177	0.14 (0.08, 0.26), <0.001	0.16 (0.07, 0.35), <0.001	0.41 (0.11, 1.50), 0.176	0.32 (0.20, 0.52), <0.001
Baseline CD4 count (cells/mmc)						
≤200	1.00	1.00	1.00	1.00	1.00	1.00
201–350	0.12 (0.05, 0.27), <0.001	0.13 (0.02, 1.04), 0.055	0.59 (0.39, 0.88), 0.010	0.34 (0.15, 0.79), 0.012	0.78 (0.29, 2.09), 0.625	1.27 (0.91, 1.76), 0.154
351–500	0.10 (0.05, 0.19), <0.001	0.20 (0.05, 0.75), 0.017	0.53 (0.35, 0.80), 0.003	0.15 (0.07, 0.33), <0.001	0.32 (0.12, 0.90), 0.031	0.89 (0.62, 1.27), 0.523
501–750	0.09 (0.05, 0.16), <0.001	0.07 (0.01, 0.58), 0.014	0.49 (0.31, 0.76), 0.002	0.16 (0.08, 0.32), <0.001	0.36 (0.13, 1.01), 0.052	1.09 (0.76, 1.56), 0.643
>750	0.07 (0.03, 0.14), <0.001	0.44 (0.09, 2.08), 0.302	0.49 (0.24, 1.02), 0.055	0.21 (0.10, 0.42), <0.001	0.29 (0.07, 1.20), 0.087	0.72 (0.38, 1.34), 0.298

